# The affinity of antigen-binding domain on the antitumor efficacy of CAR T cells: Moderate is better

**DOI:** 10.3389/fimmu.2022.1032403

**Published:** 2022-10-17

**Authors:** Rui Mao, Wanqing Kong, Yukai He

**Affiliations:** ^1^ Georgia Cancer Center, Medical College of Georgia, Augusta University, Augusta, GA, United States; ^2^ South Carolina Governors School for Science and Math, Hartsville, SC, United States; ^3^ Department of Medicine, Medical College of Georgia, Augusta University, Augusta, GA, United States

**Keywords:** Adoptive cell therapy, chimeric antigen receptors (CAR), CAR T cells, antigen-binding domain, T cell engineering, tumor immunotherapy, solid tumors

## Abstract

The overall efficacy of chimeric antigen receptor modified T cells (CARTs) remain limited in solid tumors despite intensive studies that aim at targeting multiple antigens, enhancing migration, reducing tonic signaling, and improving tumor microenvironment. On the other hand, how the affinity and engaging kinetics of antigen-binding domain (ABD) affects the CART’s efficacy has not been carefully investigated. In this article, we first analyzed 38 published solid tumor CART trials and correlated the response rate to their ABD affinity. Not surprisingly, majority (25 trials) of the CARTs utilized high-affinity ABDs, but generated merely 5.7% response rate. In contrast, 35% of the patients treated with the CARTs built from moderate-affinity ABDs had clinical responses. Thus, CARTs with moderate-affinity ABDs not only have less off-target toxicity, but also are more effective. We then reviewed the effects of ABD affinity on the biology and function of CARTs, providing further evidence that moderate-affinity ABDs may be better in CART development. In the end, we propose that a fast-on/fast-off (high *K_on_* and *K_off_*) kinetics of CART-target engagement in solid tumor allow CARTs to generate sufficient signaling to kill tumor cells without being driven to exhaustion. We believe that studying the ABD affinity and the kinetics of CART-tumor interaction may hold a key to designing effective CARTs for solid tumors.

## Introduction

Immunotherapy is now the 4^th^ pillar of cancer treatment (1, 2), and its efficacy relies on the tumor-infiltrating T cells (3), which, unfortunately, many solid tumors do not have (4). Engineering patients T cells with a T cell receptor (TCR) (5–8) or chimeric antigen receptor (CAR) (9) provides the much-needed tumor-specific T cells. CAR combines the antibody specificity and TCR signaling apparatus, which can activate T cells upon engaging with tumor surface antigen (9). The CAR-modified T cells (CARTs) thus recognize and kill tumor cells independent of MHC that is frequently downregulated, a common cause of tumor escape. CARTs have generated remarkable antitumor responses in treating hematological cancers (9–12), which results in 7 FDA-approved CARTs (13), but also ignites tremendous effort to develop solid tumor CARTs (14, 15). However, despite intensive studies, by far, the clinical efficacy of solid tumor CARTs remains limited (13, 16–18). A meta-analysis of 22 solid tumor CART trials (268 patients) reveals merely ~9% response rate (19). Evidently, the current CARTs do not work well for solid tumors. However, since 90% of cancers are solid tumors (20), investigators have been diligently working on and looking forward to a breakthrough in designing effective solid tumor CARTs.

To generate antitumor effects, CARTs need to migrate into a solid tumor mass, undergo antigen-driven activation and expansion, exert their effector function on target cells, persist sufficiently long enough to eradicate the entire tumor mass, and then form immune memory to monitor and prevent tumor relapse. Several excellent reviews (18, 21–23) have discussed the multiple strategies to improve each of these steps in the hopes of enhancing the efficacy of solid tumor CARTs. These approaches include 1) targeting multiple antigens to prevent tumor escape and to avoid off-tumor toxicity, 2) enhancing CART trafficking and infiltration into solid tumors (24–28), 3) improving the tumor microenvironment (TME) (29, 30). In addition, scientists have been studying strategies to improve CART fitness and persistence by selecting proper T cell subset (31), by reducing tonic signaling (such as utilizing 4-1BB (32) or single ITAM CD28 (33) as co-stimulatory domain), and by co-expressing C-Jun (34) or constitutive STAT5 (35). Furthermore, CAR expression under an inducible promoter (36, 37) or with a SynNotch switch (38) could diminish tonic signaling and exhaustion. These efforts have resulted in some incremental improvement. However, the overall efficacy of solid tumor CARTs still remains low. Thus, it is imperative to explore and study other components in the CAR in order to improve the efficacy of solid tumor CARTs.

A typical CAR is composed of the antigen-binding domain (ABD), hinge and transmembrane domain (TM), and intracellular signaling domain that normally consists of 1-2 co-stimulatory domains (CD) and the ζ chain (13). Each component contributes to the CART’s function and antitumor efficacy (39, 40). For example, the CD plays an important role in CART activation and persistence, and the consensus view is that CD28 generates stronger CART activation, but the 4-1BB CD renders CARTs longer persistence (32, 41). On the other hand, although the ABD is critical by rendering CAR specificity, how ABD affinity affects the activation and expansion, survival, and persistence of CARTs remained largely unknown until recently. In addition, the effect of ABD affinity on clinical efficacy of CARTs has not been studied. In this article, we will first review the response rate of solid tumor CART trials and correlate their efficacy to ABD affinity. Then, we analyze the effect of ABD affinity on CART biology and function, including activation, expansion, function, and exhaustion. In the end, we propose that CARTs with moderate-affinity ABD and fast-on/fast-off “fly-kiss” engaging kinetics will likely generate better effects in treating solid tumors.

## Correlation of ABD affinity and clinical efficacy of CARTs: Moderate is better

The ABD, most of which are the single chain variable fragment (scFv) of monoclonal antibodies (mAbs), allows CARTs to specifically bind and kill tumor cells. However, thus far, the ABD affinity was not rationally considered in most CAR designs. This is reflected by the incomplete data of ABDs, the lack of *K*_on_ and *K*_Off_, or inconsistent use of *K*_D_ (dissociation constant) and EC_50_ (half-maximal effective concentration), which will be discussed later in detail. For clarity, we refer to mAb binding strength as “affinity” (single pair of molecules) and the CAR or CART binding strength as “avidity” (multiple pairs of molecules). Over last few decades, affinity enhancement has been the main goal in antibody drug development. Approximately 100 therapeutic mAbs have been approved by the FDA (42), and many are high-affinity. Naturally, these clinically safe high-affinity mAbs, such as Cetuximab (K_D_=1.8nM) (43) and Herceptin (K_D_=5nM) (44), were used to create CARTs. High-affinity mAbs are preferred for CAR construction also because they may induce strong T cell activation and detect low levels of antigen (45–47). However, it is unclear how the ABD affinity affects the antitumor efficacy of solid tumor CARTs. Recently, we analyzed 14 solid tumor CART trials and found a trend that moderate affinity ABDs correlate to better efficacy (13). Thus, our first goal in this article is to verify the correlation of ABD affinity and clinical efficacy by expanding analysis to more solid tumor CART trials.

**Figure 1:**
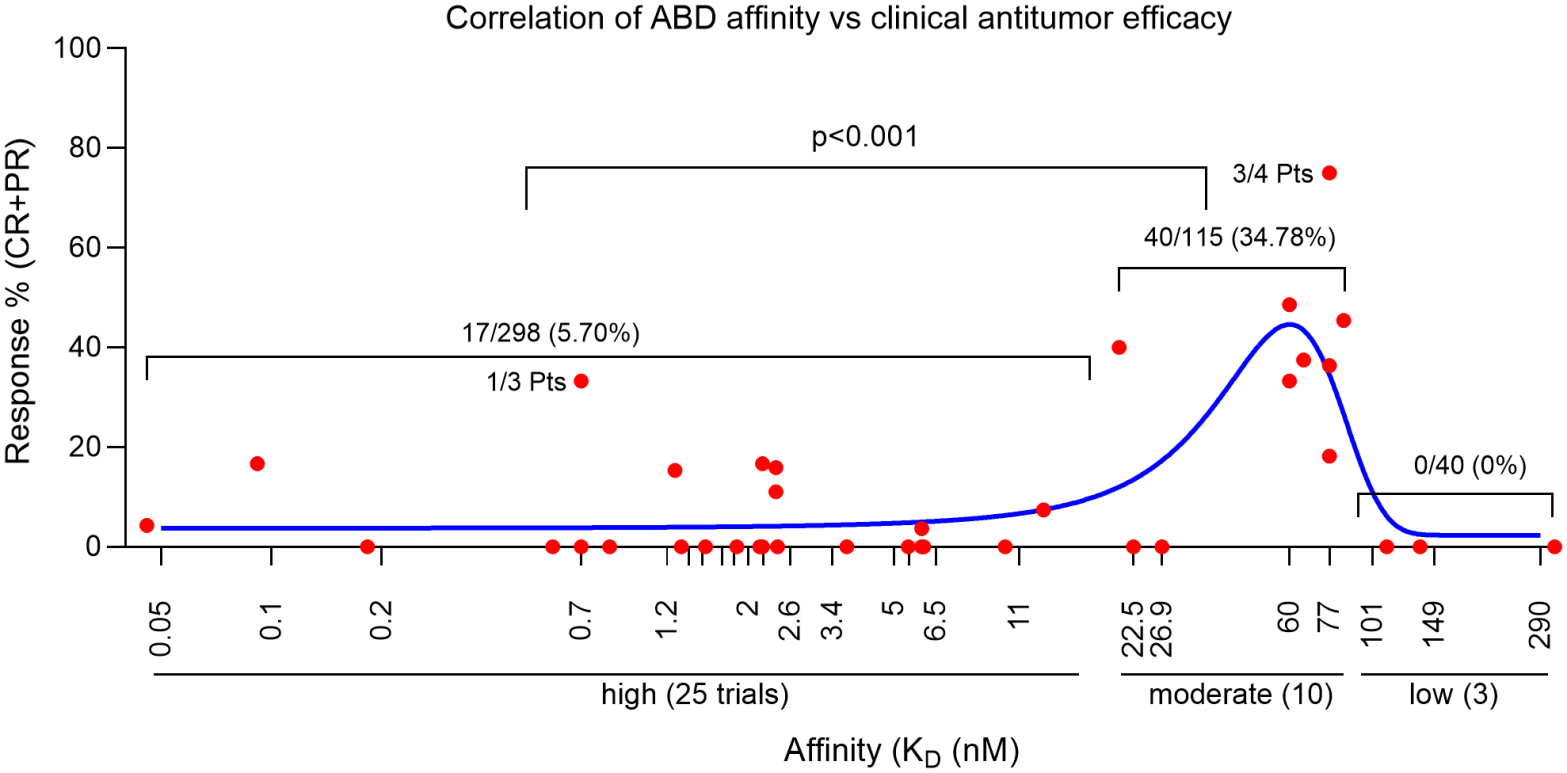
Correlation of CART clinical efficacy and their ABD affinity. Each red dot represents the % of responses of one clinical trial. The blue line is the Gaussian regression. Statistics was done with NCSS II software (Kaysville, Utah).

Based on the latest counting, there are 292 solid tumor CART trials in the world (48, 49), most of them are Phase I studies and have not completed. We were able to find 38 published solid tumor CART trials (total 453 patients). We analyzed and summarized the clinical response (partial and complete response, PR and CR) of each trial in **Table 1** and **Supplemental Table 1**. We found that, among the 453 patients in the 38 trials, 57 (12.58%) patients had PR or CR. This is seemingly higher than the response rate of ~9% reported in another meta-analysis (19), which is likely due to the latest addition of Claudin 18.2 CART trials that demonstrated 44.64% in 56 gastrointestinal (GI) cancer patients (143–145). Importantly, from the 38 CART trials, we traced back to the original CART development and found the ABD affinity (K_D_) (**Table 1** and **Supplemental Table 1**). The correlation of ABD affinity and response rate was also presented in **Figure 1**. We arbitrarily divided the ABDs as high- (K_D_<20nM), moderate- (K_D_=20-100nM), and low- (K_D_>100nM) affinity. Not surprisingly, 2/3 of the trials (25/38, 65.79%) utilized high-affinity ABDs in their CARTs. The response rate in the high-affinity group is merely 5.70% (17 out of 298 patients) (**Figure 1** and **Supplemental Table 1**). Only 9 of the 25 trials generated low response. The other 16 CARTs built from high-affinity ABDs showed no responses (the best result is stable diseases). In contrast, 8 out of 10 trials of CARTs with moderate-affinity ABDs showed an impressive response rate (18.18%-75%). The overall response rate of moderate-affinity ABD CARTs reaches 34.78% (40 out of 115 patients). Thirdly, when the ABD affinity is too low (K_D_>100nM), the CARTs demonstrated no clinical responses (**Figure 1**, **Table 1**, and **Supplemental Table 1**), suggesting that when the ABD affinity is below a certain threshold, the CARTs will not have adequate avidity to engage and kill tumor cells. These 38 trial data demonstrated that the affinity of ABDs is critical in determining the efficacy of solid tumor CARTs. ABDs with proper moderate-affinity may have the optimal engagement for CARTs to kill tumor cells inside tumor mass. Currently, there is no available data on the optimal ABD affinities in different CARs. However, it is likely that the optimal affinity of ABDs may vary among different CARTs and may depend on the engagement modes of CART-tumor cells in hematological cancers vs. solid tumors (see sections below).

**Table 1:** Summary of solid tumor CAR-T clinical trials: Affinity of antigen binding domains vs. clinical efficacy.

Target	ABD (K_D_)	ICD	*In vitro*/Preclinical	Clinical Responses
VEGF-R2	Bevacizumab (K_D_: 58pM), or Ranibizumab (46pM) (50, 51)	ζ	Anti-mouse VEGF-R2 mAb (DC101) and mouse CARTs generated no effect (52), but co-expression of IL12 regressed several mouse tumors.	NCT01218867 (Results were tabulated on the website): 1/23 PR (metastatic melanoma and renal Ca). As DC101 mAb did not recognize human VEGF-R2 (53), the Bevacizumab or Ranibizumab, or mAb from (53) (K_D_ from 0.49-1.1nM) are likely used.
CD171 (L1-CAM)	CE7: 0.1nM (54, 55)	ζ	The IgG1-Fc (hinge)CD4TM- CD3ζ CART (56) killed tumor cells and produced cytokines *in vitro*.	NCT00006480 (57): 1/6 PR (only 56days), pediatric recurrent or refractory NB, CAR-Ts disappears in a week in high tumor burden and 42 days in limited tumor burden patients.
FRα	MOv18: 0.2nM (58, 59)	FcϵRIγ	Dual allo-TCR and FRα CART inhibited tumor growth in mice (60)	NCT00019136 (61) (12 OVCA): 0 response, No tumor reduction in any of 12 patients.
Mesothelin	SS1: 0.7nM (62, 63) Epitope: AA314-375 (Beatty: WO2015090230A1)	28ζ BBζ 28-BBζ	Compared to BBζ CART, 28ζ and 28BBζ CARTs generated stronger antitumor effects (63, 64). CARTs were generated by lentivector (63) or by mRNA electroporation (64).	NCT01355965 (65) (3 MPM): 1 PR but developed anaphylaxis & cardiac arrest, due to anti-SS1 Ab (66).
				NCT01897415 (67): (6 PADC): 0 PR, 2 SD. 1 metabolic CR in the liver mets.
				NCT0215971 (68) (15 patients of MPM, PDAC, OVCA): 0 PR 11 SD. CART persisted <28days, 8 developed anti-CAR Ab.
				NCT02465983 (69): PDAC, 0/3 PR, 1SD. This is a combined CART trial of CD19 and Mesothelin (SS1 scFv) CARTs.
	M912: 1.5nM (EC_50_) (70). Converted to K_D_ (8.6-20nM) based on reference (71)	28ζ		NCT02414269 (Intrapleural local delivery of CART& PD1) (25 MPM, 1 metastatic lung Ca, and 1 metastatic breast Ca): 8 SD (among which, 2 CR) (72)
	P4 (human Ab) (73). K_D_: 1-10nM (74)	ζ 28ζ	1. P4 28z CARTs generated better effects than CD3z CARTs (74). 2. P4 CART with PD1+TCR KO (MPTK) generated much better effect than P4 CART (75).	NCT03545815 (Only MPTK CART was tested in patients (76): 15 patients (12 GI Ca and 3 other Ca): 0 PR/CR; 2 SD; CART was short lived, peaked 7-14days and undetectable after 4 weeks.
	M5 mAb (Human mAb); K_D_: 26.9nM. Epitope: aa485-572 (Beatty et al: WO2015090230A1)	BBζ		NCT03054298 (14 OVCA, MPM, lung Ca): 0 PR. Similar to SS1, M5 CART peaked D14 & disappeared after D28.
				NCT03323944 (3 PDAC): 0/3 PR (https://www.med.upenn.edu/cellicon2021/assets/user-content/documents/tanyi.pdf.
	G11 mAb: 2.35nM (77)	28ζ	Good antitumor effects in ovarian ca xenografts (77)	No clinical trial No. 3 patients of Ovarian Ca. 0/3 PR, 2/3 SD (77)
GPC3	GC33: EC_50 =_ 0.24nM (78); K_D_=1.38nM (79)	28ζ	Preclinical study showed antitumor effects (80).	NCT02395250 and NCT03146234 (13 liver Ca): 2PR (81), 1 patient survived more than 2 yrs.
	YP7 (82), EC_50_: 0.3nM	BBζ BB-28ζ	YP7-BBz CART has antitumor effects (83). But 3^rd^ gen may be toxic	NCT05003895: Started in 8/2021, Not data yet
C-Met	Onartuzumab: 1.2nM (84)	BBζ		NCT01837602 (85) (6 metastatic breast cancer) Intratumoral injection of mRNA-CAR-Ts, No response (0/6)
CEA	MFE23: 1.7nM (86)	FcϵRIγ CD3ζ	Preclinical study showed CD3z CART was better than FcϵRIγ (87)	NCT01212887 (88) (14 patients with GI Ca (metastatic). 0 PR, 7 SD, Short persistence, off-target toxicity.
	hMN14: 3.4nM (89)	28ζ	Preclinical study (90) showed 28ζ CART was better than CD3ζ CART	NCT01373047 (91): 6 patients with CEA+ liver Mets. Hepatic artery injection of CARTs with (3) or without (3) IL2 support. 1SD.
ROR1	UC-961: 2nM (92)	BBζ		NCT02706392 (93). (4 TNBC, 2 NSCLC). Decreased tumor burden at some mets, 1PR after 2 infusion (94)
GD2	Hu3F8 (95) (humanized murine 3F8 mAb) K_D_: 11nM	28-BBζ 28-27ζ	CART’s cytotoxicity diminished when repeatedly exposed to the tumor (96). CAR^hi^ Ts were depleted after co-culture with tumor cells (97).	NCT02765243 (75). (10 pediatric neuroblastoma, NB): 0 PR, 4 SD.
	murine 14.G2a (95) K_D_: 77nM	ζ	EBV-CTL with 14.G2a-CD3ζ CAR expanded maintained long-term in the presence of EBV-infected B cells (98). However, CAR-ATC (activated general T cells) did not expand by GD2.	NCT00085930: Initial report (99) found EBV-CTL transduced with CAR generated better expansion than CAR-ATC, but a later report (100) showed that CAR-ATC persists 4 yrs. Clinical outcome: 3 CR (2 sustained for > 4yrs), 1PR, 1SD out of 11 patients.
		28-OX40ζ	14g2a-28-OX40ζ CAR signaling induces sustained clonal expansion (101)	NCT01822652 (102). 11 NB: 5 SD, among the SD, 2 became CR after salvage treatment. Higher dose plus chemo and PD1 extended survival.
		28ζ, BBζ, OX40ζ 28BBζ 28OX40ζ	In tumor xenografts, the 3^rd^ gen **28BB**ζ **CARTs** showed better survival and antitumor effects (103).	NCT03373097. 5/11 PR+CR out of 11 patients, correlating to persistence of CARTs and low PMN-MDSC in blood (104).
		BBz		NCT04196413 (105): ¾ showed PR in treating DIPG
	KM8138 (Humanized KM666) (106) K_D_: 149nM	28ζ	Preclinical study showed *in vitro* killing activity and antitumor effects in mice (107)	NCT02761915 (108, 109): 0/12. No response in all 12 relapsed/refractory neuroblastoma patients, but some response in soft tissue and bone marrow disease for 3 patients.
EGFR	E10 (GenBank No: JQ306330.1) (110). It has higher affinity than 11F8 (K_D_ 2.6nM (111).	BBζ		NCT01869166 (lv-CART);1. NSCLC: 2/11 PR (2-8 mos) (112)2. Biliary Tract Ca: 1/17 CR (113)3. Pancreatic Ca: 4/16 PR (2-4mos) (114)
	No info on scFv, likely E10	BBζ	Preclinical study (115), CAR delivered by Piggybac vector	NCT03182816 (76) (Piggybac CART) NSCLC: 1/9 PR
EGFRvIII	C2173(humanized 3C10): K_D_: 101nM (116, 117) (original 3C10: 10nM)	BBζ	Some antitumor effects in human glioma xenografts in NSG mice (116)	NCT02209376 (118).No significant clinical effect (1/10 SD)
	C139 (119) K_D_: 290nM (Table 30.1 in US patent 7.628.986.B2)	28-BBζ	C139 CARTs kill target cells and produce cytokines (119). In mice, the CART generated antitumor effects in intracerebral glioma (120)	NCT01454596 (121): 0 out of 18 glioblastoma patients had responses (0/18 PR).
HER2	FRP5 mAb, KD: 6.5nM (122, 123)	28ζ	Osteosarcoma model (124); Medulloblastoma model (125)	NCT00902044: SD 4/17 (sarcoma patients) (126), 1/10 CR (metastatic sarcoma) (127, 128).
				NCT01109095: 0/17 PR, SD 3/17 (glioblastoma) (129)
	4D5 (humanized is Herceptin): 5nM. K_D_ of 4D5-28z for cells is 0.3nM (44).	28ζ 28-BBζ	Preclinical study (44) showed better persistence of 28BBζ than 28ζ CARTs	NCI-09-C-0041 (NCT00924287) 0/1Death of the patient related to off-target toxicity (130)
CA IX	G250: 2.2nM (table 1 in reference (131))	CD3ζ		DDHK9729/P00.0040C: 0/12 renal cell carcinoma (132).
PMSA	3D8: 22.5nM (133)	CD3ζ	*In vitro* study showed killing (134)	NCT01929239 (Tufts): 2/5 PR effect, last 2.5-5months (135)
	J591, K_D_: 1.83nM (136, 137)	28ζ	*In vitro* study (138), specific killing.	NCT01140373 (MSKCC): 0/7 PR, 2/7 SD, persist 2 wks (139).
		BBζ	Co-expressing dominant negative TGFRII (140): Increased proliferation and cytokine, resistance to exhaustion, persistence, and antitumor effects in human prostate cancer mouse models	NCT03089203 (Penn) (141): 0/13 PR according to RECIST. 1 patient had a 98% reduction of PSA and death due to CRS, 3 other patients have a 30% reduction of PSA
Claudin 18.2	hu8E5-2I scFv (142) EC50 (ELISA): 6.4nM. According to the conversion formula in the reference (71), the K_D_ by SPR should be 37~83nM.	28ζ	28ζ CARTs show slightly better cytotoxicity *in vitro* (142). CARTs built with hu8E5-2I CART showed good antitumor effects in treating xenografts	NCT03159819 (143):. Total 12 (11 evaluable) patients (7 Gastric Ca (GCa), 5 Pancreatic Ca(PCa)): 1CR (GCa), 3PR (2GCa, 1PCa), 5SD, 2PD
				NCT03874897 (144): GCa, 18/37 PR.
				NCT04404595 (145): Done in USA. 8 patients (5 GCa, 3 PCa). 1CR (GC), 2PR (GC), 2SD (PCa), 3PD (PCa).

PR, Partial response; CR, Complete response; SD, Stable disease; PD, Progression disease; PSA, Prostate specific antigen; PMSA, Prostate-specific membrane antigen; lv, Lentiviral vector; NSCLC, Non small cell lung carcinoma; PCa, Pancreatic cancer; PDAC, Pancreatic ductal adenocarcinoma; GCa, Gastric cancer; GI Ca, Gastrointestinal cancer; mos, Months; MPM, Malignant pleural mesothelioma; NB, neuroblastoma; OVCA, Ovary cancer

**Figure 2:**
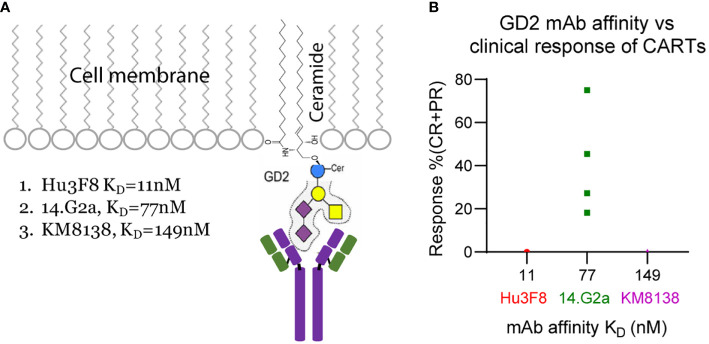
GD2 sugar moiety **(A)** and correlation of anti-GD2 mAb affinity with clinical efficacy **(B)** of the GD2 CARTs.

The 38 solid tumor CART trials in **Table 1** target different antigens and epitopes. It is known that the epitope location (relative to the cell membrane) plays an important role in deciding CART’s functions and antitumor efficacy in preclinical tumor models (27). To minimize the effects of epitope location and to analyze the correlation of ABD affinity more precisely to the antitumor efficacy of CARTs, we compared the clinical response of three GD2 CARTs. Disialoganglioside GD2, a major ganglioside, is a carbohydrate antigen expressed on the tumors of neuroectodermal origin, including melanoma, neuroblastoma, sarcoma, and small cell lung cancer (146). GD2 has a hydrophobic ceramide tail inserted into the cell membrane and a pentasaccharide moiety head on the outside of membrane (**Figure 2A**) (147). Multiple anti-GD2 mAbs are developed for cancer therapies (148), and some are approved by FDA (149). The three anti-GD2 mAbs used to develop CARTs have different affinities (**Table 1**) but target the same membrane-proximal sugar moiety (147, 150). Thus, the effect of epitope location can be neglected when the CART’s efficacy is compared. In four clinical trials using the GD2 CARTs made with moderate-affinity mAb 14.G2a (K_D_=77nM), 13 out of 37 patients had PR or CR (35.16% response rate) (**Table 1**, **Supplemental Table 1**, and **Figure 2B**). Although the 14.G2a-based GD2 CARTs in different trials utilized different CDs, they all generated good clinical responses, further suggesting that ABDs may play a deciding role in the antitumor outcome of CARTs. In contrast, the GD2 CARTs built with high-affinity Hu3F8 mAb (K_D_=11nM) or with low-affinity KM8138 (humanized KM666) mAb (K_D_=149nM) did not generate clinical response (**Figure 2B**, **Table 1**, and **Supplemental Table 1**).

The benefit of moderate-affinity ABDs in solid tumor CARTs was further demonstrated in 3 latest trials (two were in China and one was in USA) of the same Claudin 18.2 CARTs (143–145). Claudin 18.2, a member of the tight junction protein family, is considered a gastric-specific isoform with higher expression on cancers than normal tissue. Claudin 18.2 specific mAbs and CARTs are being developed to treat GI cancers. In the latest trial of Claudin 18.2 CARTs, 18 out of 37 GI cancer patients demonstrated an overall response rate of 48.64% (144). A similar response rate was also reported in other two recent trials (143, 145). The overall response rate of this Claudin 18.2 CARTs reached an impressive 44.64% in 3 trials (25 out of 56 patients). Unfortunately, the K_D_ of Claudin 18.2 mAb, 8E5, was not reported. However, investigators did measure the EC_50_ of 8E5 mAb binding to Claudin 18.2 + 293 cells, which is 49.19nM (142). After humanization and optimization, the final mAb Hu8E5-2I used in the Claudin 18.2 CARTs has EC_50_ 6.4nM for binding Claudin 18.2 + 293 cells (142), which is 20x lower than GC33 (EC_50 =_ 0.24nM) and YP7 (EC_50 =_ 0.3nM). According to a comparative study (71), the value of EC_50_ determined by ELISA is 5.76-13 folds lower than the K_D_ value measured by surface plasmon resonance (SPR). Based on this factor, we calculated the K_D_ value of Hu8E5-2I is likely between 36 to 83nM, which falls in the moderate-affinity range. We thus used the average 60nM to do the plot in **Figure 1**.

Moderate-affinity ABD is also good for blood cancer CARTs. While the original CD19 CARTs built with FMC63 mAb (K_D_=0.328nM) generated remarkable antitumor efficacy and have been approved by FDA, recent studies showed that CD19 CARTs made with a new mAb CAT with lower affinity (K_D_=14nM) generated enhanced expansion and prolonged persistence in treating refractory AML compared to the FMC63-based CD19 CART (151).

In summary, although multiple factors may contribute to CART’s function, the data analysis of 38 solid tumor CART clinical trials demonstrate that the ABD affinity is possibly the most important one in deciding the CART’s antitumor efficacy. Moderate affinity ABD not only allows CARTs to distinguish the antigen^high^ tumor cells from antigen^low^ normal cells (see section “the ABD affinity and CART’s on-target/off-tumor toxicity”), but also enable them to generate stronger antitumor efficacy. Thus, different from antibody drugs, in the CART development, moderate ABD affinity may be better.

One exception to the “moderate-affinity” role is the mesothelin targeting M5 CART. The M5 mAb is moderate affinity (K_D_: 26.9nM), but the M5 CARTs had no antitumor effects in treating multiple solid tumors (https://www.med.upenn.edu/cellicon2021/assets/user-content/documents/tanyi.pdf). Further analysis showed that the K_off_ of M5-mesothelin is low, thus the dwell time (T_1/2_) of M5-mesothelin is much longer than that of the moderate affinity mAbs of 3D8 and 14.G2a (**Table 2**). The T_1/2_ of M5-meosthelin is 613 seconds, while the T_1/2_ of 3D8-PMSA and 14.G2a-GD2 complex is 5 and 62 seconds, respectively. Thus, even though they have similar moderate affinity, their binding kinetics of targets are different (see the section of “The *K_D_*, *Kon, K_off_*, and *T_1/2_* of ABDs and their effects on CARTs”). Due to the limited examples, it remains to be verified whether the higher K_off_ (thus shorter dwell time) of ABD-antigen complex is indeed important in deciding the efficacy of solid tumor CARTs.

**Table 2:** Relationship of K_off_ and T1/2 of ABDs and clinical efficacy of CARTs.

Target	mAbs	K_on_	K_off_	K_D_ (nM)	T_1/2_ (second)	Efficacy (PR+CR)% (Responder/total patients)
PMSA	3D8 (133)	6.04e+6	1.36e-1	22.52	5	40 (2/5)
	J591 (137)	1.02e+5	1.23e-4	1.21	5,634	0 (0/20)
GD2	14.G2a (95)	1.5e+5	1.12e-2	74.67	62	30 (10/33)
	Hu3F8 (95)	9.19e+4	1.03e-3	10.4	673	0 (0/10)
	KM8138 (106)	1.14e+4	1.7e-3	149	407	0 (0/12)
Mesothelin	M5*	4.2e+4	1.13e-3	26.9	613	0 (0/17)
	SS1*	5.55e+6	5.60e-4	0.1	1,237	2.7 (1/27)

*The K_on_, K_off_, K_D_, and T_1/2_ of mAbs M5 and SS1 were from Patent: WO2015090230A1. The clinical trial reference was the same as **Table 1**.

## The effects of ABD affinity on the biology and function of CARTs

Different from conventional small molecule and antibody medicines, CARTs are living drugs, i.e., they multiply and expand, and must be alive and activated to be functional. In general, soluble antigen does not induce CART activation and expansion (152), suggesting that oligomerization of CARs on cell surface is important in CART activation although the immunological synapse of CAR is nonclassical and not well defined (153, 154). The engaging avidity between CART and target cell is determined by the ABD affinity, CAR level, and antigen level (155). In this article, we focus on the effect of ABD affinity on CART’s biology and function, such as activation, function, persistence, and antitumor effects, especially in solid tumors, where the engagement between CART and tumor is multi-dimensional, persistent, and intense.

The ABD affinity needs to reach a threshold for CARTs to have a productive engagement with tumor cells, which generates sufficient signaling to activate and expand CARTs and to kill tumor cells. An increase of ABD affinity within a range may enhance CART activation and function (156). However, ABD affinity beyond a certain level will not further enhance CART function (157), but may be harmful. The ABD affinity can affect CART biology and function in the following ways. **1)** When the CART-tumor cell engagement is too strong, the CARTs are difficult to dissociate from the killed or dying tumor cells. The occupied CARTs will be unable to re-engage with different target cells and induce serial killing of tumor cells. **2)** A strong CART-tumor cell engagement may allow CAR to nibble a piece of the target cell membrane and the associated antigen (158). This process of trogocytosis will tag the CARTs to become the target and victim of other CARTs (fratricide). Trogocytosis also cause tumor escape due to antigen loss on target cells. For example, the CD19 CARTs based on high-affinity FMC63 mAb had higher trogocytosis and fratricide than the CD19 CARTs from a lower-affinity CAT mAb (158, 159). **3)** The strong and persistent engagement of high avidity CARTs with tumor cells may drive CARTs to exhaustion and activation-induced cell death (AICD). We recently found that, compared to the CARTs with from high-affinity GC33 mAb (K_D_=1.38nM), our GPC3-specific CARTs derived from a novel moderate-affinity 8F8 mAb (K_D_=23nM) are less exhausted and less apoptotic inside tumor lesions (79). **4)** The ABD affinity affects the polyfunctionality of CARTs. Using CyTOF technology, Michelozzi et al. compared the FMC63 (high-affinity) and CAT (moderate-affinity) CD19 CARTs and found that, after engaging with CD19+ leukemia cells, the CAT CD19 CARTs contained significantly more polyfunctional T cells than the FMC63-derived CARTs (160). This suggests proper moderate-affinity ABD may allow CARTs to preserve their polyfunctionality, which is important for antitumor effect (161). Similarly, we also observed that the low-affinity 8F8 CARTs maintain better cytokines of IL2 and IFNγ production inside solid tumor lesions (79). **5)** The ABD affinity affects the formation of memory T cells. Previous studies showed that reduction of TCR functional avidity *via* lowering Lck expression (162) or by TCR downregulation (163) increased memory T cells. Similarly, our recent study (79) showed that, compared to GC33 CARTs, our 8F8 CARTs contained more memory T cells and persisted longer in the solid tumors. Importantly, the moderate-affinity 8F8 derived CARTs also maintained better function in the tumors, resulting in durable antitumor effects in treating human tumor xenografts. **6)** The ABD with reduced affinity allow CARTs to differentiate tumor cells from normal cells based on quantitative antigen difference, which will broaden the targetable tumor-associated surface antigens that can benefit tumor selectivity (164) (also seem below).

## The ABD affinity on CART’s antigen sensitivity and on-target/off-tumor toxicity

Moderate-affinity ABD may be good for CARTs to maintain function. However, lowering ABD affinity may reduce CART’s sensitivity of detecting the antigen^low^ tumor cells. For example, compared to the EGFR CARTs derived from the high-affinity Cetuximab (K_D_=1.8nM), the CARTs derived from the low-affinity Nimotuzumab (K_D_=21nM) could distinguish antigen^high^ vs. antigen^low^ target cells, but showed less control of antigen^low^ human tumor xenografts in mouse (43). Fortunately, affinity is not the only factor that affect antigen sensitivity. The affinity of TCR is much lower than CARs, but is able to detect single molecule of pMHC complex (165), while CARTs need 200 molecules of antigen for activation (166). Even with the same affinity, the sensitivity of TCR 10-100 times higher than CAR (167), suggesting that the signaling apparatus of TCR complex also play an important role in deciding the antigen sensitivity. Along this line, it was reported that manipulation of CD domain and ITAM enhanced the antigen sensitivity of CARTs (168). Thus, it is possible to lower the ABD affinity while maintaining the antigen sensitivity.

A positive side effect of losing antigen sensitivity is the reduction of on-target/off-tumor toxicity because most tumor antigens are not unique to tumor cells, but rather are the shared self-antigens that are also present in normal cells albeit at lower levels. In fact, the initial studies of utilizing low-affinity ABDs in CART development were intended to distinguish the antigen^high^ tumors from antigen^low^ normal cells to avoid off-tumor toxicity (43, 47, 169–173). Some recent preclinical *in vivo* studies further illustrated that the CARTs derived from low-affinity ABDs were indeed less toxic. Using the transgenic mice that express different levels of HER2 antigen, Castellarin et al. showed that CARTs built with low-affinity HER2 mAbs had less *in vivo* toxicity, but also generated better antitumor effects compared to high-avidity CARTs because they are less likely be trapped in the antigen^low^ normal tissues (173). In another latest report, Giardino et al. developed a pair of new GPC3-specific mAbs, GPC3-1 (K_D_=73nM) and GPC3-2 (K_D_=11nM), which could bind both human GPC3 and mouse GPC3. They demonstrated that GPC3-1 and GPC3-2 CARTs generated similar antitumor effects in mouse models. However, the low-avidity GPC3-1 CARTs demonstrated much lower toxicity in mice than the GPC3-2 CARTs (174). Thus, it is important to find an optimal moderate-affinity ABD to construct CARTs that maximize its effects on target tumor cells, while minimizing off-tumor toxicity. Different targets may need different optimal affinities. For example, in our meta-analysis of clinical trial data, we found that the CARTs built with the K_D_ of ABDs between 20-100nM generated effective CARTs (**Figure 1**). However, in the ICAM-1 targeted CARTs, the *K*_D_ of LFA binding ICAM-1 is at micromolar (*K*_D_=20µM) to generate the most effective antitumor effects with reduced toxicity in preclinical tumor model (171).

## The *K_D_*, *Kon, K_off_*, and *T_1/2_* of ABDs and their effects on CARTs

The affinity can be measured by SPR and ELISA. ELISA measures the EC_50_ (the concentration required to obtain a 50% maximum protein-ligand binding), whereas SPR measures the association (*K_on_*) and dissociation rate (*K*_off_) for the calculation of equilibrium dissociation constant *K_D_* (equal *K_off_/K_on_*), a more widely used parameter for binding affinity. Individual *K_on_* (Number/M*S) and *K*_off_ (Number/S) value can represent ligand binding kinetics much better in a time-dependent manner (175): A higher number of *K_on_* means faster ligand binding whereas a higher *K*_off_ indicates that the complex dissociates faster. As both *K_on_* and *K_off_* determined the ligand binding affinity (*K_D_*), 2 ligands with same or similar affinity (*K_D_*) might have different *K_on_* and *K_off_* value changing in the same direction (either increase or decrease), and thus show completely different binding kinetics. In this case, the ABD binding kinetics (*K_on_* and *K_off_*) may be even more important than K_D_ or EC_50_ in determining the CART’s efficacy. Another important parameter in comparing the ligand binding is the half-life of the complex, T_1/2_, which relates to *K*_off_ by the formula T_1/2_=Ln2 (0.693)/*K*_off_ (175). Thus, *T*_1/2_ indicates the stability or dwell time of the complex.

The effect of *K_on_* and *K_off_* (0r related dwell time (*T_1/2_*) of TCR-pMHC complex on T cell activation has been well-studied (165, 176, 177). If the TCR and pMHC have a fast on-rate (higher *K_on_*), the TCR-pMHC complex with a higher *K*_off_ (a short dwell time) can be highly stimulatory (165) because the pMHCs can bind and rebind the same TCR (178) or multiple TCRs (175) several times, creating an effective longer dwell time than a single TCR-pMHC encounter (165). This may contribute to the high sensitivity of TCR that can detect one pMHC complex on target cells (178). On the other hand, if the *K_on_* is low, the dissociated ligand will not easily rebind a TCR. Under such circumstances, the outcome of the TCR-pMHC engagement will likely depend on the dwell time of the TCR-pMHC complex. In other words, if the *K_on_* is low, the complex needs to be stable (lower *K*_off_) to generate sufficient signaling for activation.

A similar principle may apply to the CART-tumor engagement. The affinity (*K*_D_) of ABD-antigen engagement is in the range of pM-nM (179), which is ~3 logs lower than that of TCR-pMHC (176). Thus, the dwell time of antibody-antigen complex is in the range of hours or even day (180), much longer than that of TCR-pMHC (normally in seconds) (176). Such long stable engagement may result in persistent activation of CARTs that can drive them into exhaustion and AICD. A long engagement may not be necessary, but rather be harmful in solid tumors. Such argument is in agreement with the fact that moderate-affinity 14.G2a-based GD2 CARTs generated much better clinical efficacy than the high-affinity 3F8-based CARTs (**Supplemental Table 1** and **Figure 2B**). The anti-GD2 mAb 14.G2a has similar *K_on_* as 3F8, but has 10x higher *K*_off_ (95). Thus, the engagement dwell time of 14.G2a CARTs is 10x shorter than the 3F8 CARTs. This fast-on/fast-off “fly-kiss” mode of engagement by 14.G2a CARTs allows CARTs to have a shorter intermittent disengagement in the solid tumors. Such transient break during “off” time may rejuvenate and preserve CART function (37). Similarly, the moderate-affinity CAT mAb has similar *K_on_* as high-affinity FMC63, but has much higher *K_off_* (151), which may contribute to the formation of memory T cells and polyfunctionality of CAT CARTs (160) and durable antitumor effects (151). Thus, the dwell time and kinetics of ABD-target engagement may be more important than affinity (K_D_) in deciding the outcome of CARTs. For example, the M5 mAb has a T_1/2_ of 613 seconds (**Table 2**), which may be the reason why M5 CARTs did not generate therapeutic effects in clinical trials. In contrast, the 3D8 and 14.G2a CARTs that generated impressive clinical responses have the ABDs with higher K_off._ Their T_1/2_ is 5 and 62 seconds, respectively (**Table 2**). A high *K_on_* allow ABD bind target quickly, a higher *K_off_* may benefit for CART survival because of faster dissociation. This bind/off/rebind “fly-kiss” style of engagement may be optimal for CART to exert their function while avoiding being-driven into exhaustion, especially in solid tumor mass. Along this line of analysis, measuring the *K_on_* and *K*_off_ of the Claudin 18.2 mAb Hu8E5-2I should help verify if the fast-on/fast-off “fly-kiss” intermittent engagement mode indeed enhances CART’s antitumor efficacy. Similarly, it will be very interesting to know whether the recently developed low-affinity GPC3 specific mAb GPC3-1 (174) that has a high *K*_off_ will generate clinical efficacy in future trials.

## A fast-on/fast-off “fly-kiss” mode of engagement may be required for effective solid tumor CARTs

**Figure 3:**
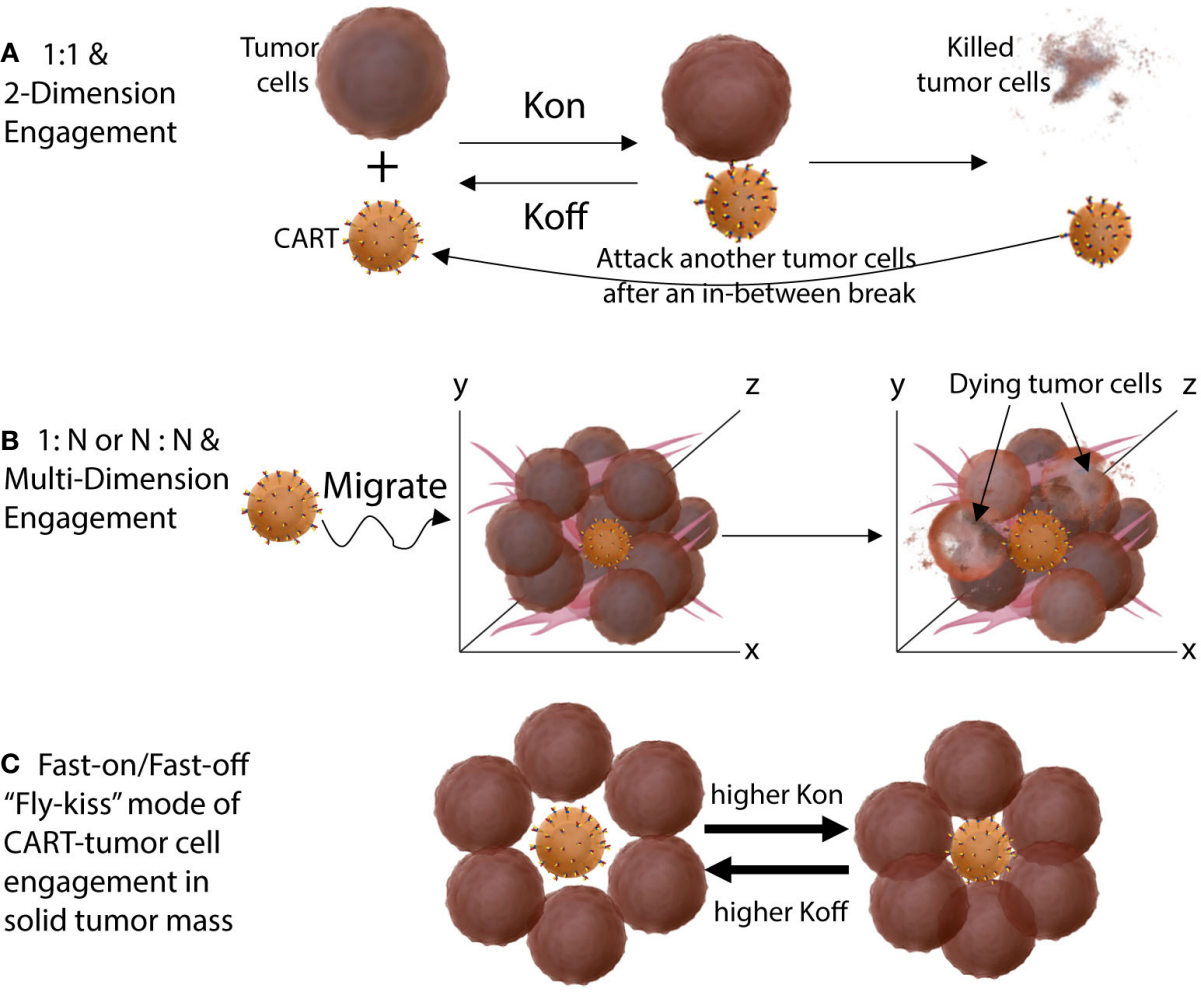
Different engagement modes of CART-tumor cells in hematological cancers **(A)** and in solid tumors **(B)**. The CARTs engage tumor cells in a solid tumor mass in 1-on-N or N-on-N multi-dimensional mode. **(C)** The fast-on/fast-off (bind-off-rebind) “fly-kiss” style of CART-tumor engagement when both the *K_on_* and *K_off_* is high. High *K_off_* allows CARTs to disengage from tumor cells even they are surrounded in solid tumors. High *K_on_* make sure that CART will re-engage tumor cells even when tumor antigen level is level.

A fundamental anatomical difference between blood cancers and solid tumors is the tumor mass, in which CART-tumor cell engagement is intense and persistent. In hematological cancers, tumor cells are in the blood and do not aggregate together to form tumor mass, and thus CARTs have immediate access to target tumor cells after infusion. Importantly, the engagement of CART-tumor cells in the blood is individualized in 1-on-1 mode and intermittent (**Figure 3**). CART can “enjoy a temporary break” after each killing before finding the next target. On the other hand, in solid tumors, CARTs first need to migrate into a tumor mass. Once CARTs infiltrate a tumor lesion, they are surrounded by tumor cells from every possible direction. Thus, the engagement of CART-tumor cells in a solid tumor is multi-dimensional 1-on-N or N-on-N mode and persistent (**Figure 3**). There is no intermittent break for the CARTs unless they can spontaneously be disengaged due to higher *K*_off_ or until the tumor mass is eliminated. Furthermore, a solid tumor has a complex extracellular matrix stroma that further restrains CART movement and aggravates the antigen assault on them. Such constant and intense engagement with antigens will drive CARTs exhaustion or AICD. Thus, due to different mode and intensity of CART-tumor engagement, the ABD affinity requirement for solid tumor CARTs is likely different from the CD19 and other blood cancer CARTs. CARTs with high-affinity ABDs will be more prone to exhaustion and AICD in solid tumors than in blood cancers.

Thus, we propose that moderate-affinity ABD and fast-on/fast-off engaging kinetics are especially necessary for solid tumor CARTs to be effective. A higher *K*_on_ of the ABD will make sure that CARTs will bind to target cell fast even when the antigen level is low; a higher *K*_off_ will allow CARTs to disengage even if they are surrounded by tumor cells in a solid tumor mass. Such a fast-on/fast-off “fly-kiss” style of engagement allows CARTs to kill tumor cells without being-driven into exhaustion and AICD. In addition, currently, we have little knowledge on how the different mode and kinetics of CART-tumor engagement may affect the epigenetics, gene expression, metabolism, and thus the fitness of CARTs. Further investigation into these mechanisms will likely help design more effective CARTs for solid tumors. We think that solid tumor CART development should focus more on the ABD affinity and the engaging kinetics of CART and tumor cells. Recently, strategies have been discussed to tune the ABD affinity for better and effective CART development (181) even though there is no obvious approach to select ABDs with particular *K*_on_ and *K*_off_ yet. This intentional and rational design of CARs with tuning ABD affinity and binding dynamics in mind will likely generate more effective solid tumor CARTs that can potentially match the remarkable success observed in hematological cancers.

## Author contributions

RM and YH analyzed the clinical responses of 38 clinical trials and searched the ABD affinity in the CARTs. WK drew the CART-tumor engagement mode presented in **Figure 3**. All three authors wrote the manuscript together and approved the submitted version.

## Acknowledgments

The CART research work in YH’s laboratory is partially funded by Paceline Cancer Research Award grant from Georgia Cancer Center, Augusta University. We thank Dr. Ramses Sadek for performing the statistical analysis of the clinical trial data. We also thank current and former members for their contributions in improving the antitumor efficacy of engineered T cells.

## Conflict of interest

The authors declare that the research was conducted in the absence of any commercial or financial relationships that could be construed as a potential conflict of interest.

## Publisher’s note

All claims expressed in this article are solely those of the authors and do not necessarily represent those of their affiliated organizations, or those of the publisher, the editors and the reviewers. Any product that may be evaluated in this article, or claim that may be made by its manufacturer, is not guaranteed or endorsed by the publisher.
